# Supplementary material for: Validating an Instrument for Direct Patient Reporting of Distress and Chemotherapy-Related Toxicity among South African Cancer Patients
Source: Cancers (Basel). 2021 Dec 24;14(1):95. doi: 10.3390/cancers14010095 (PMC8750185; doi:10.3390/cancers14010095)
Supplement: Supplementary file 1 [file cancers-14-00095-s001.zip › cancers-1424686-supplementary.pdf]

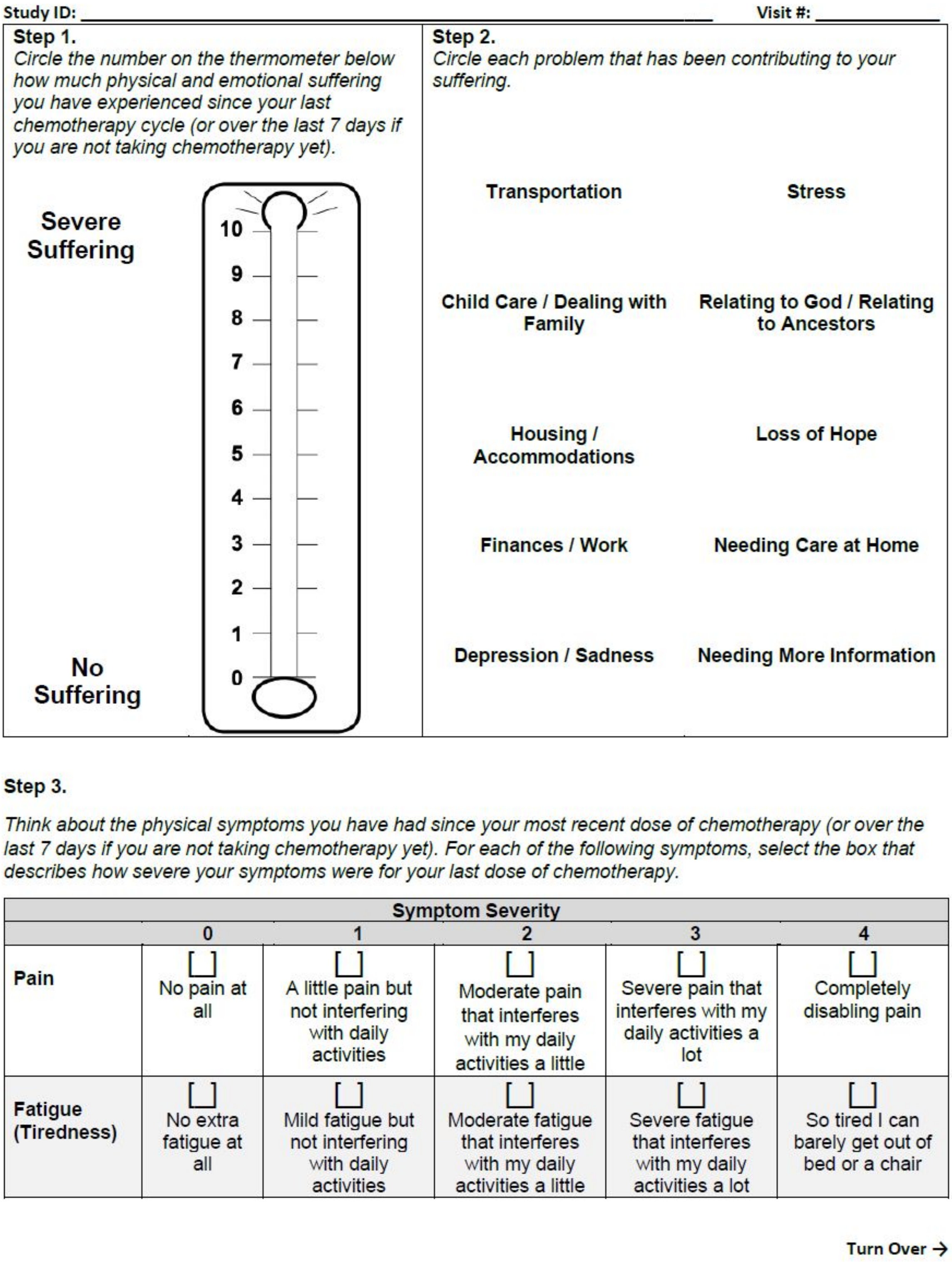

Study ID: ______________________ Visit #: ____________

Step 1.
Circle the number on the thermometer below how much physical and emotional suffering you have experienced since your last chemotherapy cycle (or over the last 7 days if you are not taking chemotherapy yet).

Step 2.
Circle each problem that has been contributing to your suffering.

**Transportation**	**Stress**
**Child Care / Dealing with Family**	**Relating to God / Relating to Ancestors**
**Housing / Accommodations**	**Loss of Hope**
**Finances / Work**	**Needing Care at Home**
**Depression / Sadness**	**Needing More Information**

Step 3.

Think about the physical symptoms you have had since your most recent dose of chemotherapy (or over the last 7 days if you are not taking chemotherapy yet). For each of the following symptoms, select the box that describes how severe your symptoms were for your last dose of chemotherapy.

	Symptom Severity				
	0	1	2	3	4
**Pain**	[ ] No pain at all	[ ] A little pain but not interfering with daily activities	[ ] Moderate pain that interferes with my daily activities a little	[ ] Severe pain that interferes with my daily activities a lot	[ ] Completely disabling pain
**Fatigue (Tiredness)**	[ ] No extra fatigue at all	[ ] Mild fatigue but not interfering with daily activities	[ ] Moderate fatigue that interferes with my daily activities a little	[ ] Severe fatigue that interferes with my daily activities a lot	[ ] So tired I can barely get out of bed or a chair

Turn Over →

Figure S1. Front page of the Patient Reported Symptoms – South Africa instrument.

Study ID: ______________________________ **Visit #:** ____________

**Fever**	[ ] No fever at all	[ ] Fever one time that went away on its own		[ ] Repeated fever and had to take antibiotics	[ ] Fever requiring hospitalization
**Difficulty Breathing (Shortness of Breath)**	[ ] No difficulty breathing at all	[ ] Difficulty breathing with heavy work	[ ] Difficulty breathing with light work	[ ] Difficulty breathing with daily activities like dressing or bathing	[ ] Difficulty breathing when resting in bed or a chair
**Cough**	[ ] No cough at all	[ ] Mild cough, improved with simple medicines	[ ] Moderate cough that interferes with my daily activities a little	[ ] Severe cough that interferes with my daily activities or sleep a lot	
**Mouth Sores or Dry Mouth**	[ ] No mouth symptoms	[ ] Mild mouth sores / dryness, no medications needed	[ ] Moderate mouth sores / dryness, using medications but eating normally	[ ] Severe mouth sores / dryness, cannot eat normally	[ ] Mouth sores / dryness requiring hospitalization
**Nausea/Loss of Appetite**	[ ] No nausea or loss of appetite at all	[ ] Nausea or less appetite but eating normally	[ ] Nausea or less appetite and not eating or drinking enough		[ ] Eating so little that I needed a drip or hospitalization
**Vomiting**	[ ] No vomiting at all	[ ] Vomiting, but only 1 time each day	[ ] Vomiting, 2 to 5 times in a day	[ ] Vomiting more than 5 times in a day	[ ] Vomiting so much that I needed a drip or hospitalization
**Diarrhoea**	[ ] No extra bowel movements (number 2)	[ ] 1 to 3 extra bowel movements (number 2) in a day	[ ] 4 - 6 extra bowel movements (number 2) in a day	[ ] More than 6 extra bowel movements (number 2) in a day	[ ] Diarrhea requiring hospitalization
**Constipation**	[ ] No constipation at all	[ ] Sometimes constipation	[ ] I cannot have a bowel movement (number 2) without medications	[ ] Constipation that interferes with my daily activities a lot	[ ] Constipation requiring hospitalization
**Burning or "Pins & Needles" in Hands and Feet**	[ ] No burning / "pins & needles" in hands or feet	[ ] Mild burning / "pins & needles", but not interfering with daily activities	[ ] Moderate burning / "pins that interferes with my daily activities a little	[ ] Severe burning / "pins & needles" that interferes with my daily activities a lot	

Turn Over →

Figure S2. Back page of the Patient Reported Symptoms – South Africa instrument.

Table S1. Pearson Correlations Between PRS-SA Symptom Item Severity and QLQ-C30 Functional Scale Scores.

	**QLQ-C30 Functional Scales**									
	**Physical (PF2)**		**Role (RF2)**		**Emotional (EF)**		**Cognitive (CF)**		**Social (SF)**	
**PRS-SA Symptom**	**r**	**p-value**	**r**	**p-value**	**r**	**p-value**	**r**	**p-value**	**r**	**p-value**
**Pain**	0.38	<0.0001	0.33	<0.0001	0.35	<0.0001	0.28	<0.0001	0.26	<0.0001
**Fatigue**	0.57	<0.0001	0.46	<0.0001	0.51	<0.0001	0.43	<0.0001	0.45	<0.0001
**Fever**	0.23	<0.0001	0.13	<0.0001	0.18	<0.0001	0.24	<0.0001	0.09	0.04
**Dyspnea**	0.45	<0.0001	0.34	<0.0001	0.29	<0.0001	0.27	<0.0001	0.26	<0.0001
**Cough**	0.22	<0.0001	0.21	<0.0001	0.18	<0.0001	0.16	<0.0001	0.13	<0.0001
**Oral Mucositis**	0.29	<0.0001	0.25	<0.0001	0.31	<0.0001	0.22	<0.0001	0.25	<0.0001
**Nausea**	0.41	<0.0001	0.39	<0.0001	0.39	<0.0001	0.31	<0.0001	0.38	<0.0001
**Vomiting**	0.30	<0.0001	0.24	<0.0001	0.22	<0.0001	0.18	<0.0001	0.24	<0.0001
**Diarrhea**	0.06	0.19	0.08	0.06	0.11	0.01	0.08	0.06	0.13	0.002
**Constipation**	0.30	<0.0001	0.22	<0.0001	0.28	<0.0001	0.28	<0.0001	0.22	<0.0001
**Peripheral Neuropathy**	0.35	<0.0001	0.26	<0.0001	0.42	<0.0001	0.42	<0.0001	0.29	<0.0001
